# Application of microalgae reduces the incidence of bacterial wilt in flue-cured tobacco by altering soil properties and microbial community characteristics

**DOI:** 10.3389/fmicb.2026.1931562

**Published:** 2026-09-30

**Authors:** Feng Tian, Min Fan, Yihua Quan, Xinyu Hu, Xinyao Liu, Xiaomin Tang, Lei Yang, Zhenhua Wang, Qiang Li

**Affiliations:** 1 College of Agriculture, Hunan Agricultural University, Changsha, Hunan, China; 2 Baise Municipal Tobacco Monopoly Bureau of Guangxi Zhuang Autonomous Region, Baise, Guangxi, China; 3 Zhangjiajie Branch of Hunan Tobacco Company, Zhangjiajie, Hunan, China; 4 China Tobacco Hunan Industrial Co., Ltd., Changsha, Hunan, China

**Keywords:** flue-cured tobacco, microalgal amendment, microbial community structure, soil chemical properties, topological indices

## Abstract

**Introduction:**

To elucidate the mechanisms by which microalgae reduce tobacco bacterial wilt incidence through modifying soil properties and microbial community structure.

**Methods:**

Flue-cured tobacco (K326) was used as the test plant. Three microalgal suspension treatments—T1 (14 mL·L^−1^), T2 (28 mL·L^−1^), and T3 (56 mL·L^−1^)—and a water control (CK) were established. Tobacco yield, bacterial wilt incidence, and soil chemical properties were measured. Soil bacterial abundance and microbial diversity were analyzed using quantitative real-time PCR (qPCR) and Illumina MiSeq sequencing.

**Results:**

1. Compared with CK, yield in T1, T2, and T3 increased by 12.2%, 17.2%, and 14.8%, respectively, while disease incidence at maturity decreased by 43.7%, 18.9%, and 71.9%, respectively; 2. Pathogen abundance at maturity in T1, T2, and T3 decreased by 74.1%, 90.7%, and 90.7%, respectively, relative to CK, and soil pathogen abundance was significantly positively correlated with disease indices; 3. Microalgae suppressed soil pathogen abundance and disease incidence by increasing soil pH and dissolved organic carbon while decreasing available potassium and available phosphorus; 4. Compared with CK, bacterial abundance at maturity in T1, T2, and T3 increased by 12%, 23%, and 33%, respectively, and biocontrol functional genera (e.g., Sphingomonas, Bacillus) were enriched; 5. Microalgae reshaped the bacterial co-occurrence network by enhancing its modularity, and this highly modular network structure significantly suppressed pathogen proliferation.

**Discussion:**

Microalgae suppressed pathogens and reduced disease incidence by modulating soil chemical properties, increasing bacterial diversity, enriching biocontrol functional genera (e.g., Sphingomonas, Bacillus), and optimizing network topology. Under the experimental conditions, microalgae application at 28 mL·L^−1^ achieved the best performance, with a yield increase of 17.2% and a pathogen abundance reduction of 90.7%.

## Graphical Abstract

**Figure:**
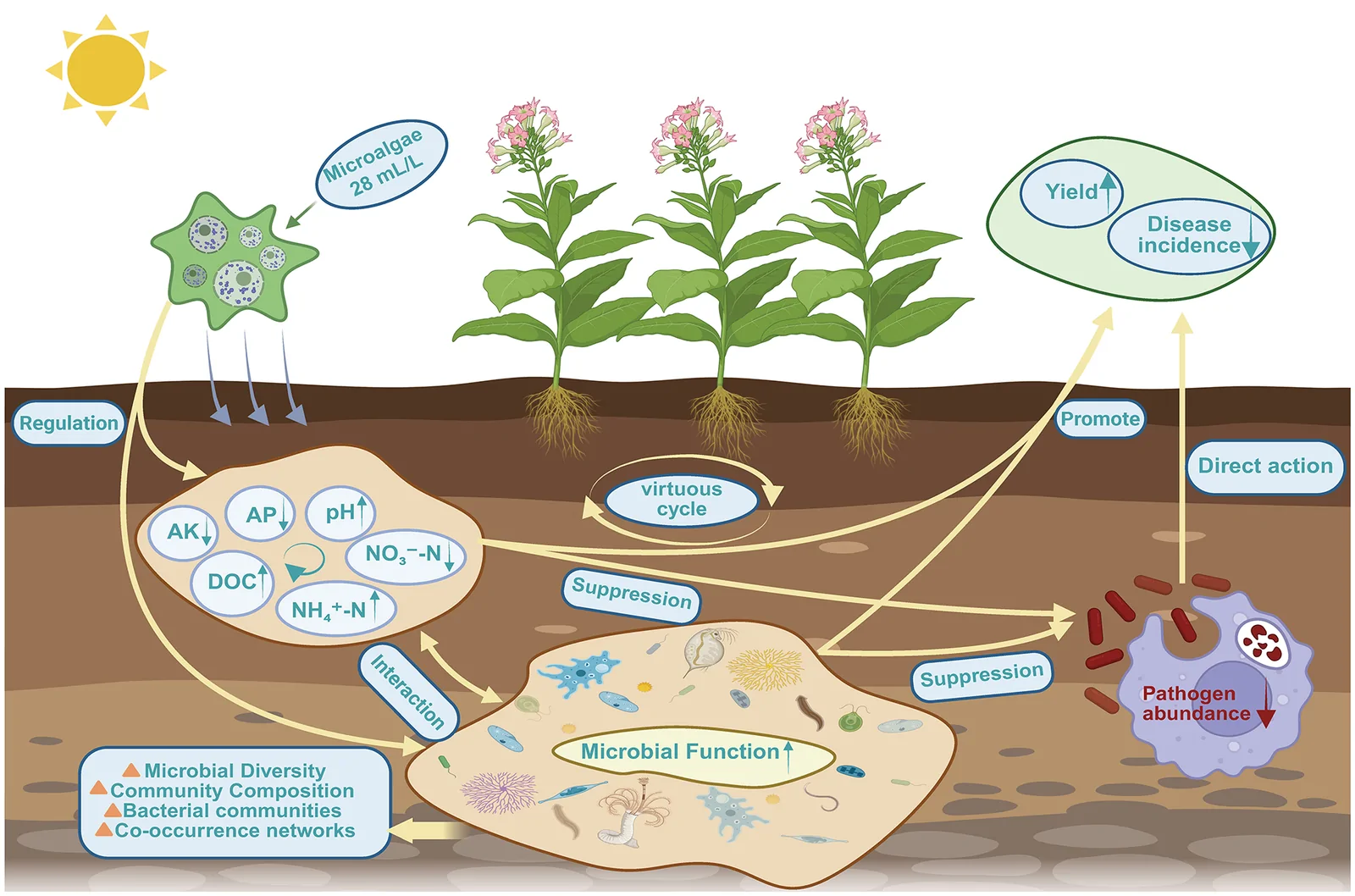

## 1 Introduction

Tobacco (*Nicotiana tabacum L*.), a globally significant economic crop, plays a vital role in supporting agricultural economies and increasing farmers' income in many regions. In major tobacco-producing provinces of China, such as Yunnan and Guizhou, its cultivation has become a core pillar of the rural economy. However, the sustainable development of this industry is severely constrained by tobacco bacterial wilt, a devastating disease caused by the soil-borne pathogen *Ralstonia solanacearum*. This pathogen is characterized by a wide host range, high survival capability, and complex transmission pathways (Jiang et al., 2017). Current management strategies for controlling tobacco bacterial wilt face significant limitations. Agronomic practices such as crop rotation and soil disinfection often fail to eradicate the pathogen, which persists in soil for extended periods, and long rotation cycles can be impractical (Ziedan, 2022); While chemical control offers short-term efficacy, it risks inducing pathogen resistance and leads to contamination of soil and groundwater with chemical residues, contradicting the principles of ecological agriculture (Sang et al., 2019); Biological control utilizing antagonistic microbes such as *Bacillus* and *Pseudomonas* is often hampered by inconsistent performance under variable soil conditions, hindering its large-scale (Jiao et al., 2020).

In recent years, research on modulating soil microecology to enhance crop disease resistance has garnered considerable attention, The application value of microalgae in agricultural ecosystems has been preliminarily demonstrated (González-Pérez et al., 2022; Ramakrishnan et al., 2023). Microalgae can promote soil nutrient cycling, influence soil microorganisms, and thereby contribute to plant growth promotion, biological control, and soil stabilization (Bibi et al., 2024); Through photosynthesis, they fix inorganic carbon and nitrogen, converting them into slow-release organic nutrients (e.g., polysaccharides, amino acids), This process significantly enhances fertilizer use efficiency, potentially achieving the goal of maintaining yield while reducing chemical fertilizer input. For example, compared to chemical fertilizer alone, the application of de-oiled microalgal biomass as a biofertilizer in potted rice effectively increased plant height, tiller number, and grain yield, thereby reducing the need for synthetic fertilizers (Nayak et al., 2019). Successful applications in crops such as rice and tomato have been reported (Gonçalves et al., 2023; Song et al., 2024); Furthermore, microalgae secrete phytohormones (e.g., indole-3-acetic acid, gibberellins) that promote root development and enhance stress tolerance in plants, Their metabolites (e.g., phycocyanin, polyunsaturated fatty acids) also serve as high-quality carbon sources for soil microorganisms, indirectly optimizing the structure of the soil micro-ecosystem (Ling et al., 2014), These multifaceted properties provide a theoretical basis for employing microalgae in the control of bacterial wilt.

The homeostasis of the soil micro-ecosystem relies heavily on the synergistic interaction between soil chemical properties and the microbial community. Soil pH, dissolved organic carbon (DOC), and the contents of nitrogen, phosphorus, and potassium not only directly affect crop growth but also indirectly determine the proliferation potential of pathogens by regulating microbial niche competition and functional differentiation (Yang et al., 2023). Soil pH is a key driver of microbial community structural divergence. An increase of one pH unit can elevate bacterial diversity by 15–20%, and such communities of higher diversity are more likely to suppress pathogen expansion through resource competition (Fierer, 2017); Competition for scarce micronutrients such as iron and manganese among rhizosphere microorganisms is a core mechanism regulating the invasion of *Ralstonia solanacearum*, This competitive process is profoundly influenced by the coupled relationship between soil DOC content and microbial community structure (Singh et al., 2025). Furthermore, topological features of microbial co-occurrence networks (e.g., modularity, clustering coefficient, average path length), which serve as important indicators of community functional stability, have become a crucial means to evaluate soil health mechanisms, Their associations with soil chemical factors and their regulatory roles in disease occurrence are central to this assessment (Liu et al., 2023).

Based on the research gaps outlined above, we hypothesize that microalgae suppress disease by restructuring the interactive network of “soil properties + microbial community characteristics,” thereby creating a soil micro-ecosystem unfavorable to pathogens. Through a field experiment, we aimed to (i) evaluate the effects of microalgae application on the incidence of bacterial wilt and tobacco yield; (ii) examine its impact on the abundance of *R. solanacearum*; (iii) analyze changes in soil properties and microbial community structure; and (iv) integrate the findings to propose a comprehensive mechanism underlying disease suppression. Our work aims to provide a theoretical basis and technical reference for the application of microalgae in the green control of soil-borne diseases.

## 2 Materials and methods

### 2.1 Experimental methods and design

#### 2.1.1 Experimental site and materials

The field experiment was conducted in May 2020 in Sangzhi County (29°18′ N, 110°02′ E), Zhangjiajie City, Hunan Province. The initial soil nutrient status (0-20 cm depth) of the experimental site was as follows: available phosphorus (AP), 41.23 mg·kg^−1^; available potassium (AK), 83.44 mg·kg^−1^; ammonium nitrogen (NH_4_^+^-N), 20.34 mg·kg^−1^; nitrate nitrogen (NO_3_^−^-N), 1.63·mg kg^−1^; pH, 6.75; and dissolved organic carbon (DOC), 65.89 mg·kg^−1^.

The test tobacco cultivar was flue-cured tobacco K326, with seeds provided by the College of Agronomy, Hunan Agricultural University. The microalgal stock suspension was supplied by Gengtianxia Biotechnology Co., Ltd., Hunan Province, and mainly comprised nitrogen-fixing cyanobacteria, Chlorella pyrenoidosa, and Tolypothrix tenuis, with a viable cell count of 3 × 106 CFU mL^−1^. The suspension was transported and stored at −4 °C under refrigeration, and diluted with water to the desired concentrations prior to use.

#### 2.1.2 Experimental design

A standard flue-cured tobacco field was selected. Three microalgal suspension treatments—T1 (14 mL·L^−1^), T2 (28 mL· L^−1^), and T3 (56 mL· L^−1^)—were established, with water irrigation serving as the control (CK). Each plant received 300 mL of the corresponding suspension, applied at 10 days after transplanting. The experiment was arranged in a completely randomized block design with three replicates per treatment, totaling 12 plots. Each plot comprised seven ridges, with 17 plants per ridge, amounting to 119 plants per plot. Nutrient management and cultivation practices followed the tobacco production technical standards of Zhangjiajie City.

#### 2.1.3 Soil sample collection

Soil samples (0–20 cm) were collected before transplanting using the five-point sampling method to serve as background samples for determining baseline soil nutrient status. Soil samples were taken once at the rapid growth stage and once at the maturity stage. Rhizosphere soil was obtained by gently brushing off the soil tightly adhering to the roots with a small brush, with three replicates collected from each plot. After removing impurities such as plant debris and stones, the soil was thoroughly homogenized. One portion was stored at −20 °C for the analysis of pH, dissolved organic carbon(DOC), nitrate nitrogen(NO_3_^−^–N), ammonium nitrogen(NH_4_^+^–N), available phosphorus(AP), and available potassium(AK) in air-dried samples from both stages. The other portion was stored at −80 °C and used as fresh soil for 16S rRNA gene sequencing and pathogen quantification.

### 2.2 Disease surveys and yield measurements

The grading and investigation of tobacco diseases followed the Chinese national standard method. The incidence and disease index of bacterial wilt were calculated based on the following criteria.

The disease severity rating scale for bacterial wilt was:

0 = no symptoms; 1 = one leaf wilted; 3 = two or three leaves wilted; 5 = all leaves except the top leaves wilted; 7 = all leaves wilted; 9 = stem collapsed or plant dead.

Disease incidence (%) = *n*′ / *N* × 100, where “n′” is the number of infected plants, and “*N*” is the total number of plants investigated.

Disease index = ∑ (*r* × *n*) / (*N* × 9) × 100, where n is the number of infected plants, “*r*” is the disease severity grade, and “*N*” is the total number of plants investigated.

For yield determination, 30 tobacco plants were randomly tagged per plot before harvest, individually harvested and flue-cured, and weighed after curing. Yield was calculated on a per-hectare basis (kg ha^−1^) according to a planting density of 16,500 plants ha^−1^.

### 2.3 Determination of soil chemical properties

Soil chemical properties were analyzed according to established methods Bao, 2000. Specifically: soil pH was measured using a pH meter (PHSJ-5T, Leici, China); ammonium nitrogen (NH_4_^+^–N) and nitrate nitrogen (NO_3_^−^–N) were extracted with 2 mol/L KCl solution and their concentrations determined using a flow injection analyzer (AA3, SEAL Analytical, Germany); soil dissolved organic carbon (DOC) was extracted with 0.5 mol/L K_2_SO4 for 30 min, filtered, and then measured using a dissolved organic carbon analyzer (Analytik Jena, Germany); available phosphorus (AP) content was determined by the sodium bicarbonate extraction–molybdenum antimony anti-colorimetric method; available potassium (AK) content was determined by the ammonium acetate extraction–flame photometry method.

### 2.4 Soil microbial DNA extraction and quantitative real-time PCR (qPCR) analysis

Soil microbial DNA was extracted using the cetyltrimethylammonium bromide (CTAB) method (Osawa et al., 2021). Briefly, 0.5 g of freeze-dried and ground soil sample was weighed and processed according to the standard procedure. The quality and concentration of the extracted DNA were checked by 1% agarose gel electrophoresis and a UV-Vis spectrophotometer (ND-1000, NanoDrop Technologies, Germany).

Quantitative PCR (qPCR) targeting the bacterial 16S rDNA was performed using the primer set 1369F and 1492R, A standard curve was generated using ten-fold serial dilutions of a plasmid containing the 16S rDNA target, The amplification efficiency was 100%~105%, with an R^2^ of 0.997. For the quantification of Ralstonia solanacearum, specific primers (forward, 5′-GAACGCCAACGGTGCGAACT-3′; reverse, 5′-GGCGGCCTTCAGGGAGGTC-3′) were used, The corresponding standard curve was constructed using ten-fold serial dilutions of a plasmid containing the target sequence from R. solanacearum, This qPCR assay had an amplification efficiency of 95%~107% and an R^2^ of 0.996.

### 2.5 Illumina MiSeq high-throughput sequencing and data processing

Purified DNA samples were sent to Shanghai Majorbio Bio-Pharm Technology Co., Ltd. for paired-end sequencing on an Illumina MiSeq platform, The resulting paired-end sequence data were subjected to bioinformatic analysis. Using the UPARSE algorithm, all effective tags from the samples were clustered into operational taxonomic units (OTUs) at a 97% similarity threshold. After basic quality control, a total of 1,117,392 high-quality bacterial sequences were obtained from the 24 soil samples, These sequences were clustered into 22,398 bacterial OTUs, which were subsequently used to calculate alpha diversity indices for each sample.

Statistical significance of differences among treatments for all measured indicators was assessed using Duncan's multiple range test in SPSS software (version 26.0)(*p* ≤ 0.05), In the figure, the error bars in all tables represent standard deviation, and statistical tests were performed using three replicates for each treatment. Taxonomic annotation was performed using the RDP Classifier (version 22) against the SILVA (version 132) database. Pearson correlation analysis was conducted to examine the relationships among soil physicochemical properties, microbial community characteristics, and disease incidence. Differences in microbial community composition were analyzed using non-metric multidimensional scaling (NMDS), and the significance of these compositional differences was tested using the ‘vegan' package (version 2.6.4) in R software (version 4.2.2). Microbial co-occurrence networks were constructed using the ‘WGCNA' (version 1.72.1) and ‘igraph' (version 1.5.1) packages in R and visualized using Gephi software (version 0.10.1). Topological feature indices of the networks were calculated using the ‘ggClusterNet' package (version 0.1.0) in R. Finally, summary figures were created using Adobe Illustrator (2025).

## 3 Results

### 3.1 Microalgae application enhances tobacco yield and reduces soil pathogen abundance

As shown in Figure 1A, during the rapid growth stage, compared with CK, the disease incidence in T1, T2, and T3 decreased by 9.46%, 48.05%, and 54.43%, respectively, and the disease index decreased by 45.31%, 63.10%, and 66.46%, respectively. At the maturity stage, compared with CK, the disease incidence in T1, T2, and T3 decreased by 43.64%, 18.88%, and 71.97%, respectively, and the disease index decreased by 69.14%, 33.13%, and 79.42%, respectively. These results indicate that microalgae treatments reduced both the incidence and disease index of bacterial wilt to varying extents. As shown in Figure 1C, significant differences were observed among treatments. Compared with CK, the yield of T1, T2, and T3 increased by 12.2%, 17.2%, and 14.8%, respectively, suggesting that microalgae effectively controlled bacterial wilt, thereby ensuring yield formation in flue-cured tobacco, with the most pronounced yield enhancement observed in T2.

**Figure 1:**
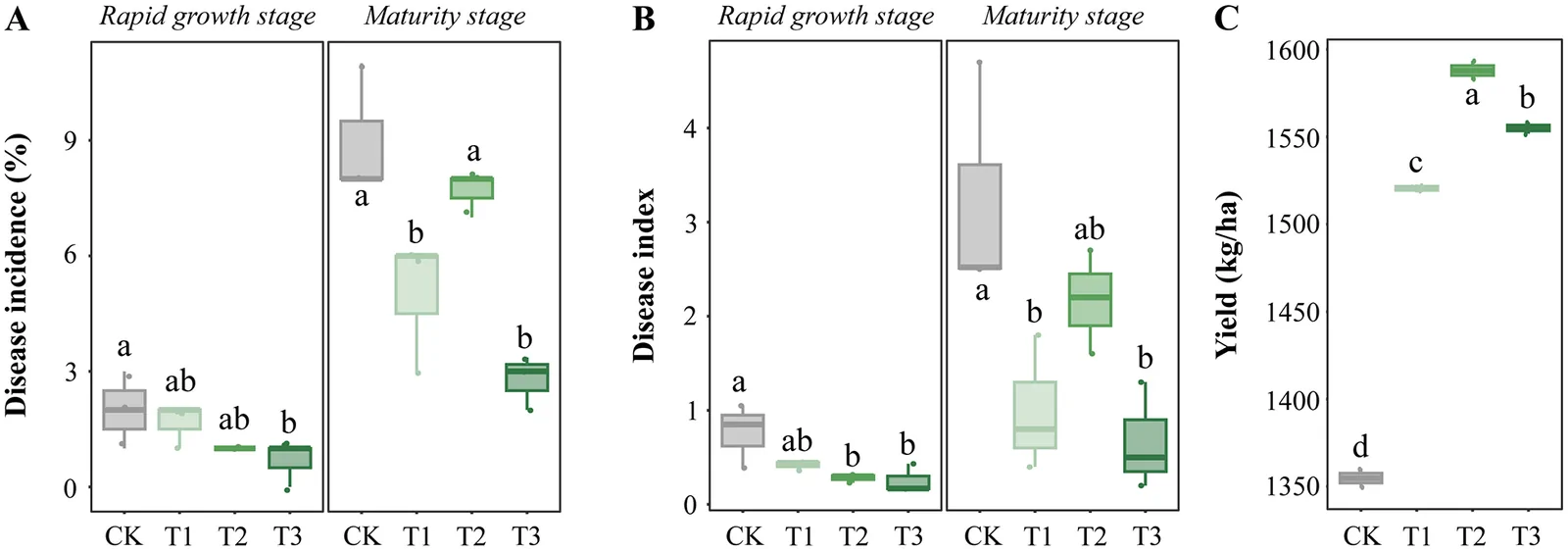
Growth conditions of tobacco under different treatments. Disease incidence **(A)**, disease index **(B)**, and yield **(C)** of tobacco under different treatments. The upper and lower boundaries of each box indicate the 75th and 25th percentiles, respectively. The dot inside the box represents the repetition of each treatment. Different letters indicate significant differences at *P* ≤ 0.05.

In summary, microalgae application significantly improved flue-cured tobacco yield by reducing the incidence and disease index of bacterial wilt across different growth stages, with the suppressive effect being more significant during the maturity stage, These phenotypic results provide supportive evidence for employing microalgae to regulate soil properties and microbial communities to control tobacco bacterial wilt.

As shown in Figure 2A, during the rapid growth stage, compared with CK, pathogen abundance in T1, T2, and T3 decreased by 20.75%, 44.78%, and 90.79%, respectively. At the maturity stage, pathogen abundance in T1, T2, and T3 decreased by 74.1%, 90.7%, and 90.7%, respectively, with T2 and T3 exhibiting more pronounced long-term suppression. As shown in Figure 2B, both disease incidence and disease index of bacterial wilt were significantly positively correlated with soil pathogen abundance at both growth stages, indicating that higher pathogen abundance was associated with a higher disease incidence.

**Figure 2:**
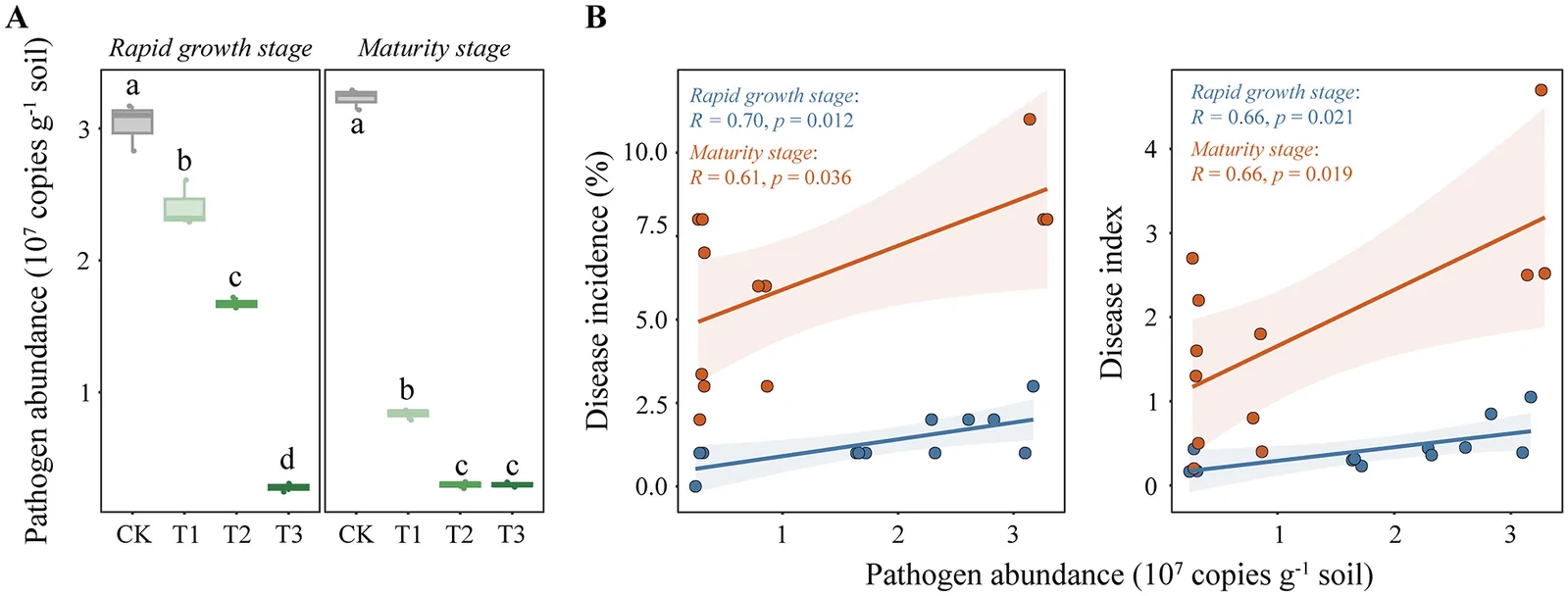
Abundance of soil pathogens under different treatments. Abundance of soil pathogens **(A)** and its relationship with tobacco disease incidence and disease index **(B)**. The upper and lower boundaries of each box indicate the 75th and 25th percentiles, respectively. The dot inside the box represents the repetition of each treatment. Different letters indicate significant differences at *P* ≤ 0.05.

### 3.2 Microalgae reduce disease incidence by altering soil chemical properties, bacterial diversity, and community composition

Microalgae treatments significantly altered soil chemical properties, including modulating nitrogen forms, increasing dissolved organic carbon (DOC), raising soil pH, and reducing available potassium (AK) and available phosphorus (AP). As shown in Figures 3A–F, At the maturity stage, compared with CK, DOC content in T1, T2, and T3 increased by 6.72%, 3.19%, and 15.27%, respectively; pH increased by 0.65%, 0.60%, and 1.66%, respectively; AK decreased by 7.18%, 18.31%, and 8.69%, respectively; and AP decreased by 1.23%, 4.93%, and 20.20%, respectively. As shown in Figure 3G, nitrate nitrogen (NO_3_^−^–N) showed a highly significant positive correlation with both disease incidence and disease index of bacterial wilt. In contrast, ammonium nitrogen (NH_4_^+^–N) was highly significantly negatively correlated with these two disease parameters. Dissolved organic carbon (DOC) and pH were highly significantly negatively correlated with pathogen abundance, whereas available potassium (AK) and available phosphorus (AP) were significantly positively correlated with pathogen abundance.

**Figure 3:**
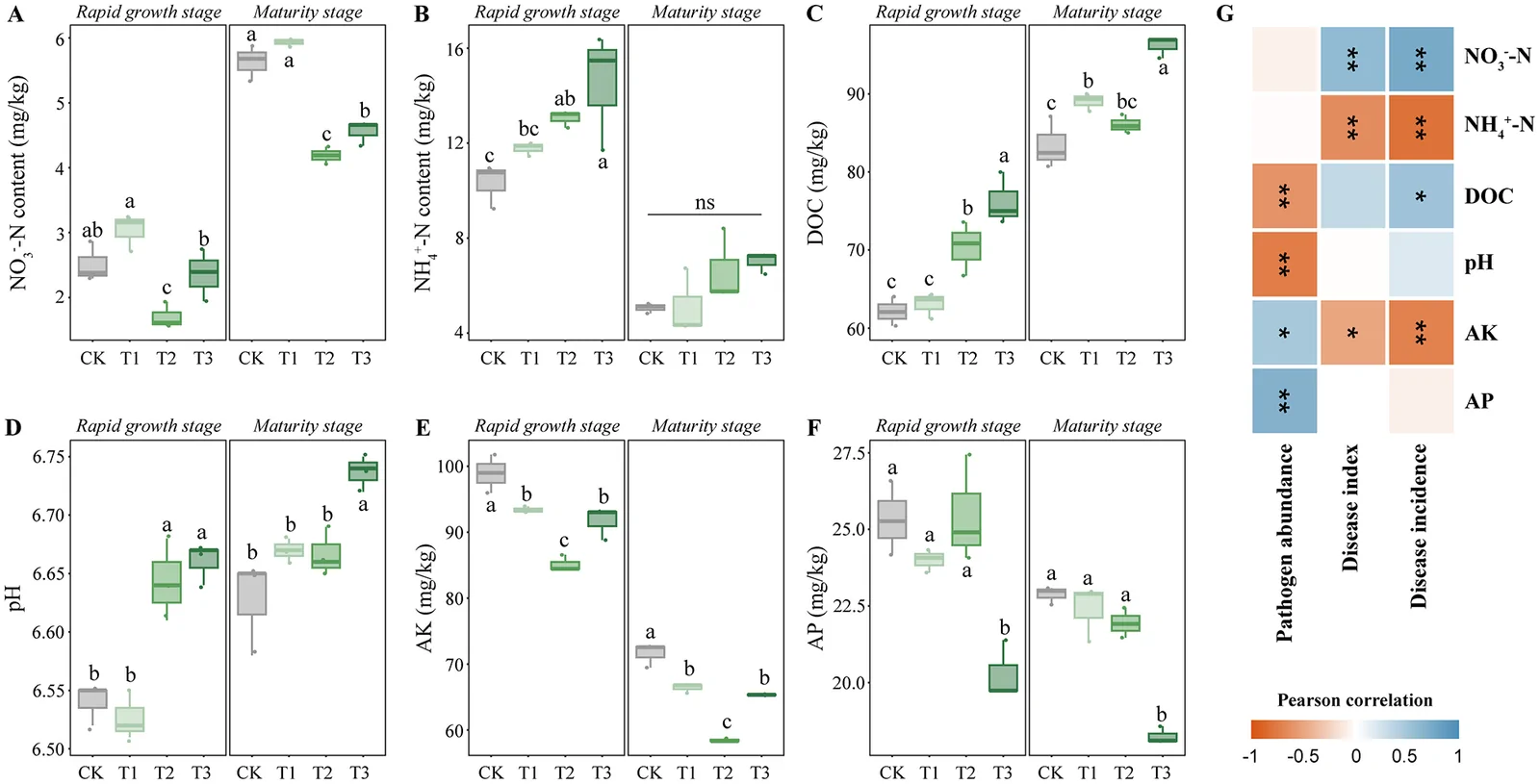
Soil chemical properties under different treatments. Soil ${\text{NO}}_{3}^{-}$-N **(A)**, ${\text{NH}}_{4}^{+}$-N **(B)**, DOC **(C)**, pH **(D)**, AK **(E)**, and AP **(F)** content under different treatments. The upper and lower boundaries of each box indicate the 75th and 25th percentiles, respectively. The dot inside the box represents the repetition of each treatment. Different letters indicate significant differences at *P* ≤ 0.05. Pearson correlation between soil properties and tobacco disease incidence, disease index, and soil pathogen abundance **(G)**. Significance levels are as follows: *P ≤ 0.05; **P ≤ 0.01; and ***P ≤ 0.001.

Microalgae application significantly increased soil bacterial abundance during both the vigorous growth and maturity stages, with abundance positively correlating with application rate (Figures 4A–G). The Shannon index (Figure 4C) indicated that microalgae treatment significantly enhanced soil bacterial diversity at the vigorous growth stage, while diversity varied with growth progression into the maturity stage. Non-metric multidimensional scaling (NMDS) analysis (Figure 4D) revealed that growth stage was a key factor shaping bacterial community composition (R^2^ = 0.26, *p* = 0.001), The treatment factor exhibited explanatory power approaching significance (R^2^ = 0.12, *p* = 0.094), suggesting a trend toward differentiation in community composition among treatments. A significant interactive effect between treatment and growth stage was also observed (R^2^ = 0.15, *p* = 0.017), As shown in Figures 4E and 4F, microalgae application significantly altered soil bacterial community composition in both stages, with a more pronounced effect during maturity.

**Figure 4:**
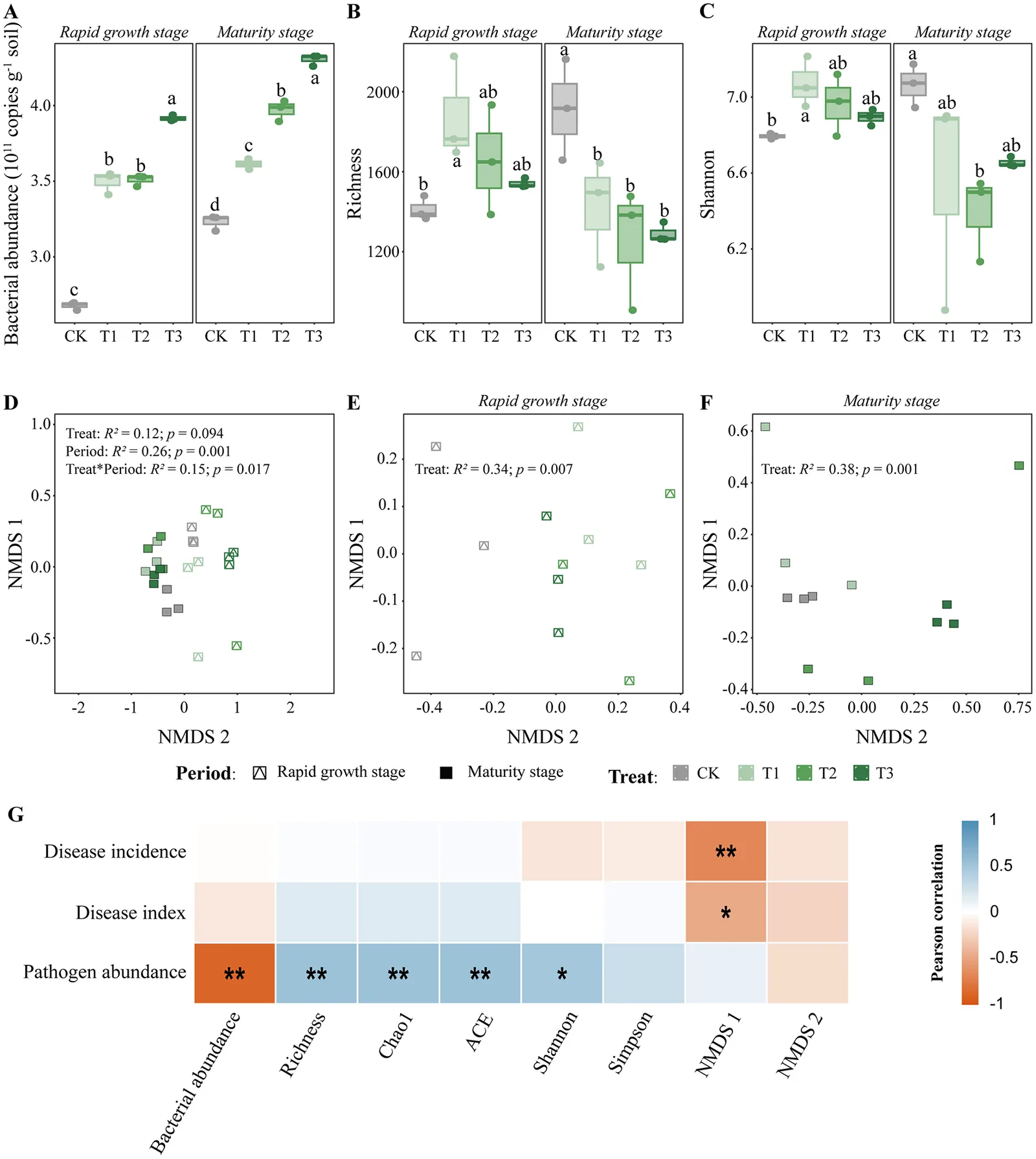
Soil bacterial diversity and community composition under different treatments. The abundance **(A)**, richness **(B)**, and shannon **(C)** of bacteria. The upper and lower boundaries of each box indicate the 75th and 25th percentiles, respectively. The dot inside the box represents the repetition of each treatment. Different letters indicate significant differences at *P* ≤ 0.05. Non-metric multidimensional scaling (NMDS, based on Bray-Curtis distances, showing the community composition of bacteria **(D)** in rapid growth stage **(E)** and maturity stage **(F)**. Pearson correlation between bacterial diversity and community composition and tobacco disease incidence, disease index, and soil pathogen abundance **(G)**. Significance levels are as follows: *, *P* ≤ 0.05; **, *P* ≤ 0.01; and ***, *P* ≤ 0.001. In the correlation analysis, microbial community composition **(G)** was represented by the first (NMDS1) and second (NMDS2) axes of the non-metric multidimensional scaling ordination (Jiang et al., 2024; Jiao et al., 2018). *P ≤ 0.05; **P ≤ 0.01; and ***P ≤ 0.001.

To further clarify the regulatory effects of soil chemical properties on the bacterial community, Pearson correlation analysis was performed to elucidate their associations with bacterial abundance, richness, diversity indices (Chao1, ACE, Shannon, Simpson), and community composition (NMDS1 and NMDS2). As shown in Figure 5, the results demonstrated that changes in nitrate nitrogen (NO_3_^−^–N) and ammonium nitrogen (NH_4_^+^–N) contents significantly drove the differentiation of bacterial community structure along the NMDS1 axis, identifying them as key nitrogen factors shaping soil bacterial community composition. Dissolved organic carbon (DOC) promoted soil bacterial proliferation (increased abundance) and diversity (elevated Shannon index), and also reshaped bacterial community structure by driving compositional differentiation along NMDS1. An increase in soil pH (a shift toward alkalinity) significantly enhanced bacterial richness. Available potassium (AK) exhibited a dual regulatory effect, suppressing bacterial abundance while promoting diversity and community differentiation. A decrease in available phosphorus (AP) significantly increased soil bacterial abundance.

**Figure 5:**
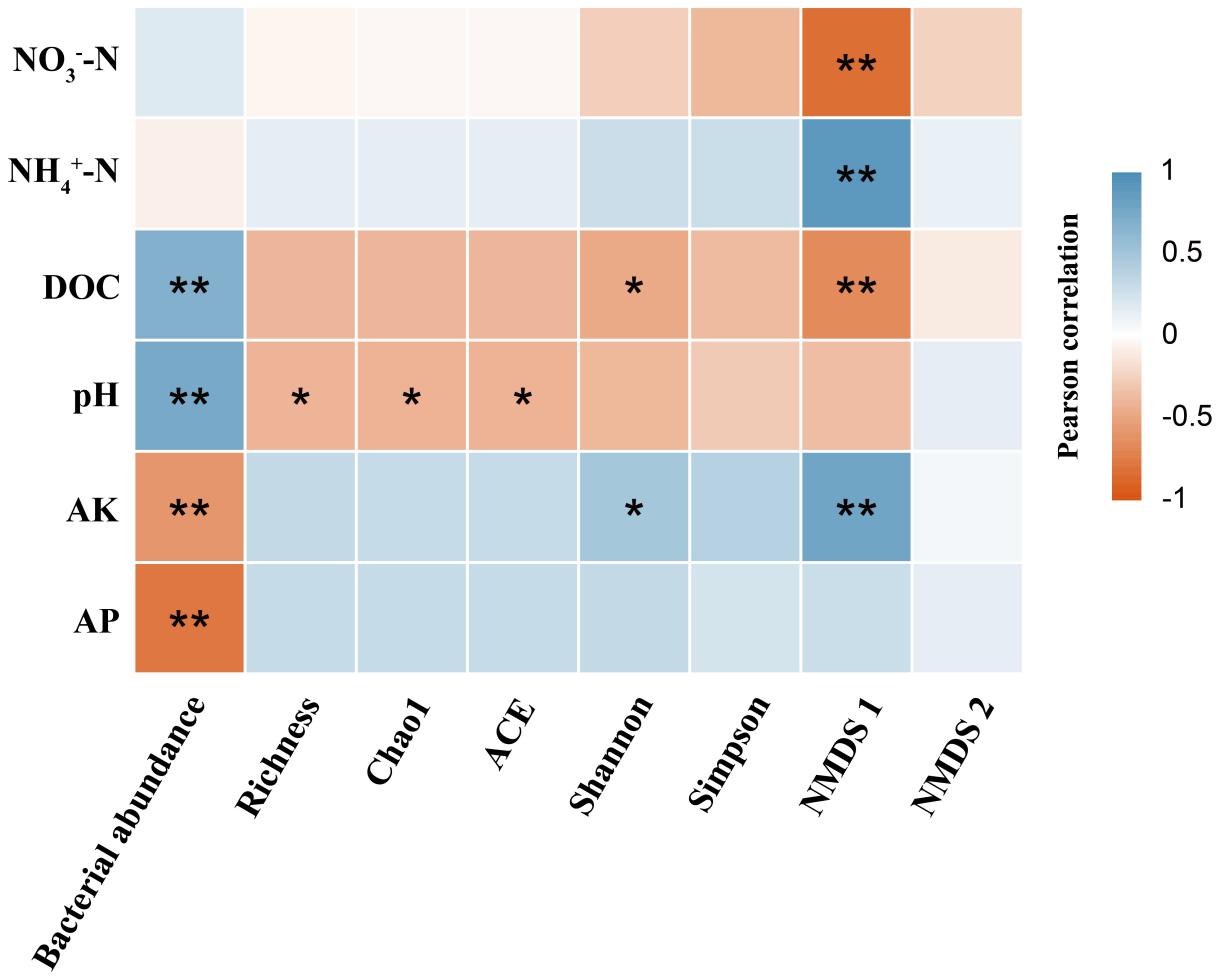
The relationship between soil chemical properties and bacterial diversity and community composition. Significance levels are as follows: *P ≤ 0.05; **P ≤ 0.01; and ***P ≤ 0.001.

### 3.3 Microalgae reduce disease incidence through interactions between soil chemical properties and dominant bacterial taxa

To elucidate the regulatory effects of microalgae treatments on soil bacterial community composition, we analyzed the relative abundance distribution of bacterial taxa at the phylum and genus levels across treatments. As shown in Figure 6A, during both the rapid growth stage and the maturity stage, the composition of dominant bacterial phyla differed significantly among treatments and between growth stages. The core dominant phyla included *Chloroflexi, Actinobacteriota, Acidobacteriota*, and *Proteobacteria*. Different microalgae treatments (T1, T2, and T3) specifically modulated the relative abundance of these dominant phyla. For instance, during the rapid growth stage, T3 significantly altered the relative abundance of *Proteobacteria*, whereas T2 markedly enriched *Acidobacteriota*. These structural differences at the phylum level reflect the regulatory potential of microalgae treatments on ecological processes such as soil carbon and nitrogen cycling and microbial interactions. Notably, *Proteobacteria* are involved in diverse nutrient metabolisms, *Acidobacteriota* are closely associated with soil carbon decomposition, and *Actinobacteriota* serve as important sources of biocontrol microorganisms.

**Figure 6:**
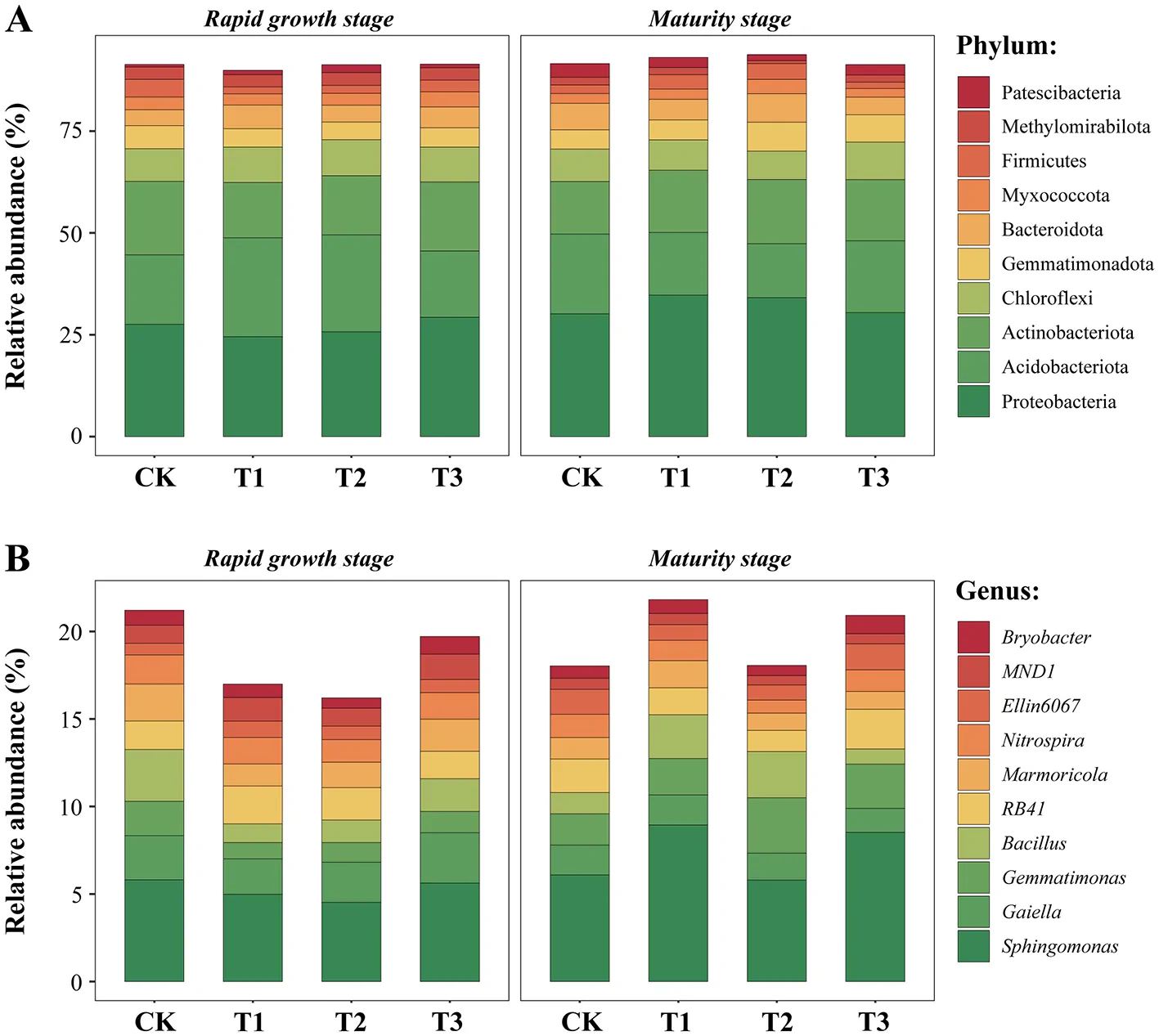
Profile of bacterial communities under different treatments. Relative abundance of soil bacterial phylum **(A)** and genus **(B)** level across treatments.

The genus level represents a key taxonomic rank for inferring bacterial community function. The abundance variations of functional genera among treatments indicate the disease-suppressive and nutrient-regulating mechanisms of microalgae. As shown in Figure 6B, Bacillus and *Sphingomonas* are *biocontrol* functional genera, and their relative abundance was significantly increased by microalgae treatments during both the rapid growth stage and the maturity stage. *Nitrospira* (associated with nitrification) was significantly enriched in T3 during the rapid growth stage, while *Gemmatimonas* (involved in carbon metabolism) exhibited higher abundance in T2 and T3.

To further clarify the regulatory roles of specific soil bacterial taxa in flue-cured tobacco bacterial wilt, Pearson correlation analysis was performed to examine the relationships among disease incidence, disease index, pathogen abundance, and the dominant bacterial phyla (Figure 7A) and genera (Figure 7B). At the phylum level, *Methylomirabilota* showed a significant negative correlation with both disease incidence and disease index; In contrast, *Patescibacteria* exhibited a significant positive correlation with these disease parameters, indicating that lower disease incidence was associated with its reduced abundance. As shown in Figure 7A, the relative abundance of *Methylomirabilota* in the T2 was lower than in other treatments during the vigorous growth stage; At the genus level, *Nitrospira* showed a significant positive correlation with soil pathogen abundance, suggesting that a lower level of its enrichment could reduce pathogen density. Consistently, Figure 7B shows that the relative abundance of *Nitrospira* in T2 was lower than in other treatments during the vigorous growth stage; *MND1* was significantly negatively correlated with both disease incidence and disease index.

**Figure 7:**
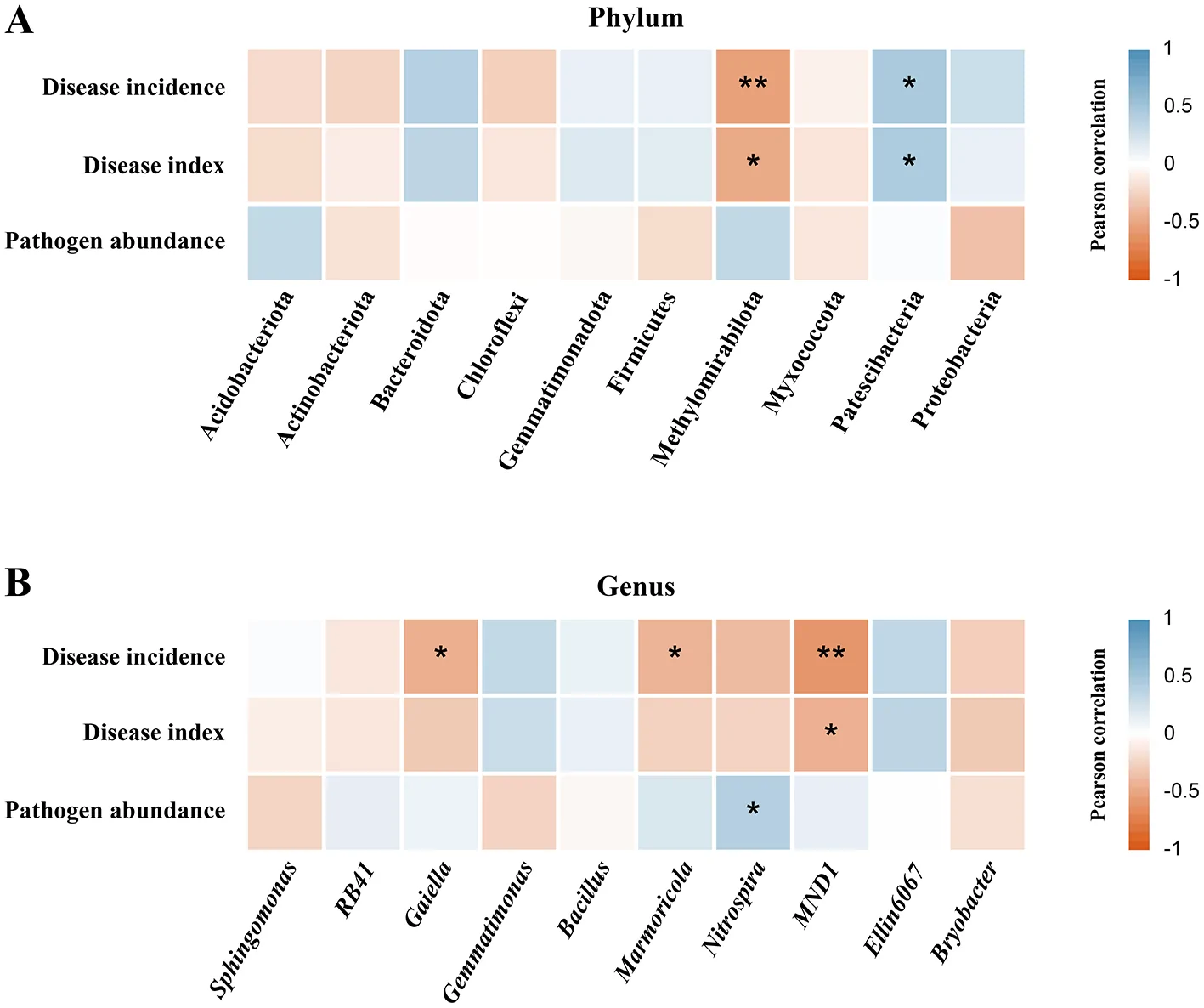
Relationship between tobacco disease incidence, disease index, and soil pathogen abundance and dominant bacterial taxa. Pearson correlation between relative abundance of soil bacterial phylum **(A)** and genus **(B)** level and tobacco disease incidence, disease index, and soil pathogen abundance. Significance levels are as follows: *P ≤ 0.05; **P ≤ 0.01; and ***P ≤ 0.001.

As shown in Figure 8, Pearson correlation analysis revealed significant correlations between soil chemical properties and both dominant bacterial phyla and functional genera. By altering soil chemical properties—such as regulating nitrogen forms, DOC, pH, and available nutrient levels—microalgae application directionally modulated the relative abundance of dominant bacterial phyla and genera. On the one hand, it suppressed the abundance of disease-promoting bacterial taxa (at both phylum and genus levels) that were positively correlated with disease, On the other hand, by optimizing the chemical environment (e.g., elevating DOC, adjusting pH), it facilitated the enrichment of disease-suppressive functional genera.

**Figure 8:**
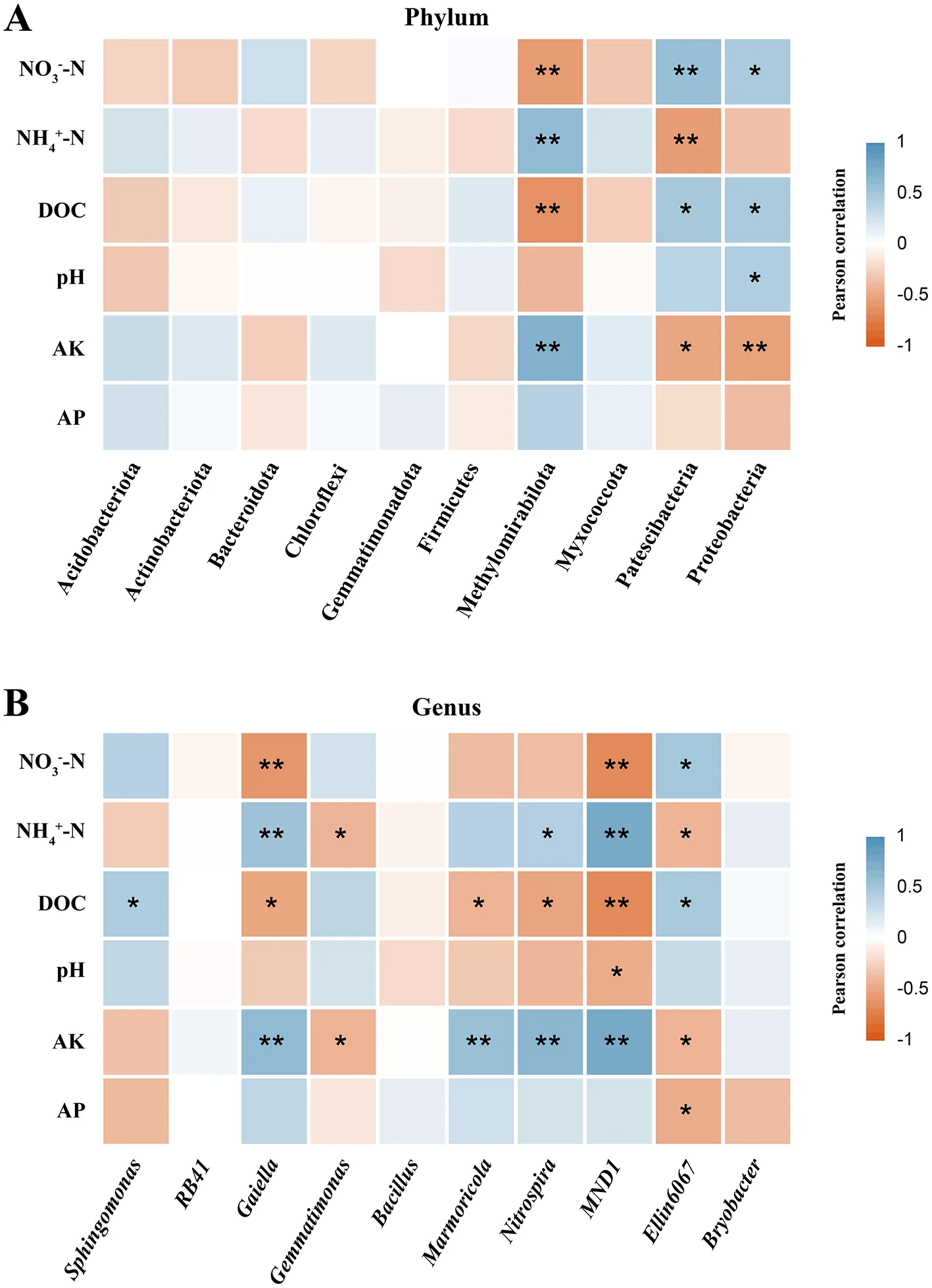
The relationship between soil chemical properties and dominant bacterial taxa. Pearson correlation between relative abundance of soil bacterial phylum **(A)** and genus **(B)** level and soil chemical properties. Significance levels are as follows: *P ≤ 0.05; **P ≤ 0.01; and ***P ≤ 0.001.

### 3.4 Interactions among bacterial co-occurrence networks, topological features, and soil chemical properties

To reveal the regulatory effects of microalgae treatment on the interactive relationships within the soil bacterial community, co-occurrence network analysis was conducted to decipher potential ecological associations among bacteria. This analysis was combined with an examination of correlations between key topological features and disease indicators, yielding the following results:

Visualization of the networks (Figure 9A) showed significant differences in node distribution and edge connection density among treatments (CK, T1, T2, T3), The CK network exhibited a dispersed node distribution, with interaction connections showing characteristics of localized clustering. In contrast, networks from microalgae-treated soils displayed more uniform node distributions and interactions that tended toward “global connectivity,” This indicates that microalgae application reshaped bacterial co-occurrence relationships, enhancing both the interaction strength and connectivity within the community. As shown in Figure 9B, key topological parameters—including the number of edges (Num. edges), number of vertices (Num. vertices), edge density, average degree, and mean clustering coefficient—were significantly lower in T2 and T3 than in CK at the maturity stage, This suggests that microalgae treatment simplified the network structure and reduced redundant interactions at maturity, shifting the community interaction pattern from localized clustering toward a more globally connected state, thereby enhancing the functional efficiency and stability of the community.

**Figure 9:**
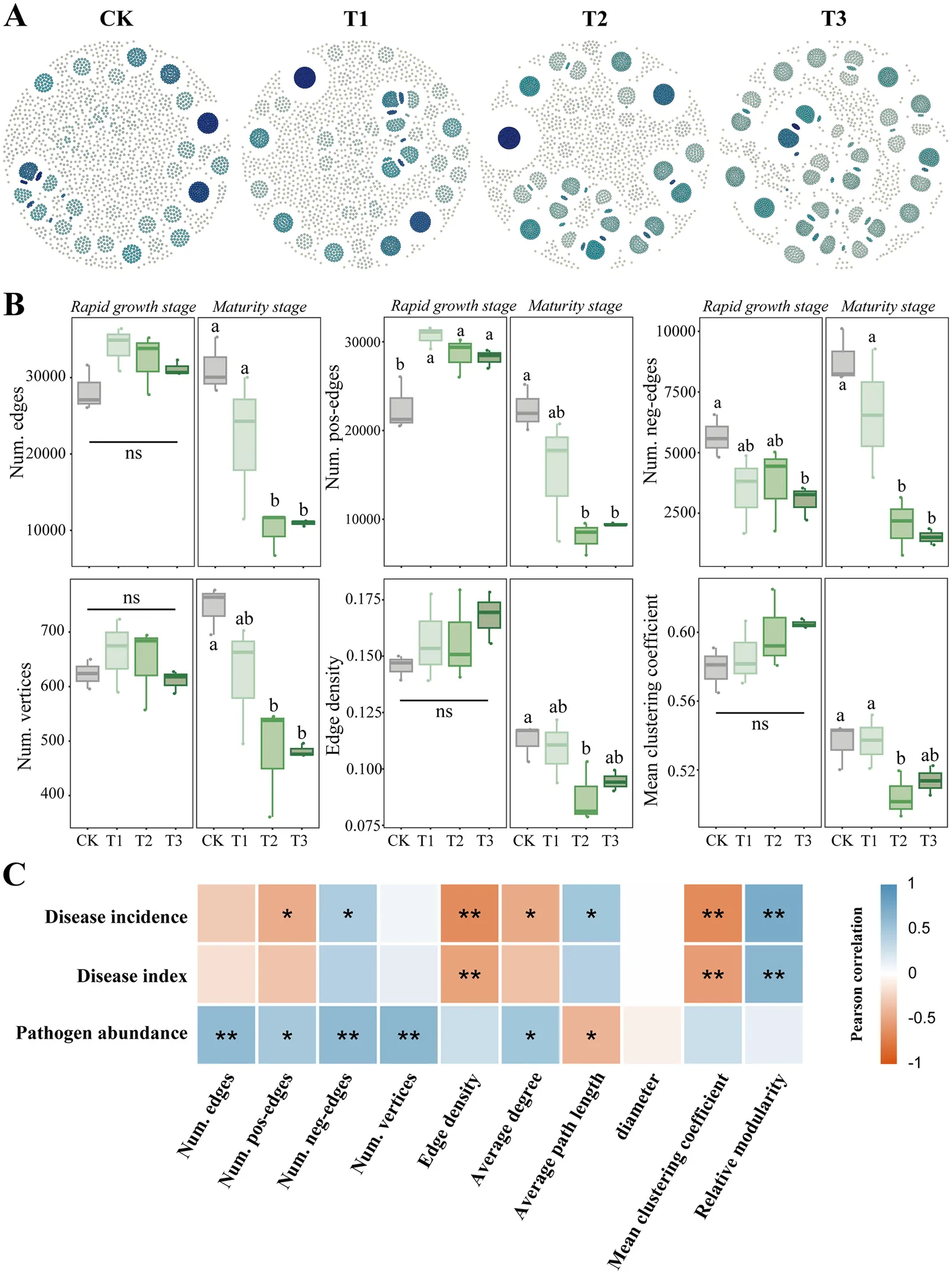
Bacterial co-occurrence networks and its topological features. Bacterial co-occurrence networks under different treatments **(A)**. The topological features of co-occurrence networks under different treatments **(B)**. The upper and lower boundaries of each box indicate the 75th and 25th percentiles, respectively. The dot inside the box represents the repetition of each treatment. Different letters indicate significant differences at *P* ≤ 0.05. Pearson correlation between topological features and tobacco disease incidence, disease index, and soil pathogen abundance **(C)**. Significance levels are as follows: *P ≤ 0.05; **P ≤ 0.01; and ***P ≤ 0.001.

Pearson correlation analysis (Figure 9C) revealed significant correlations between key network topological features and disease incidence, disease index, and pathogen abundance. Specifically, pathogen abundance showed significant positive correlations with most topological indices, This indicates that more complex network structures—characterized by higher numbers of edges (Num.edges), vertices (Num.vertices), and negative edges (Num.neg-edges)—were associated with greater pathogen abundance and, consequently, lower soil disease suppressiveness; Num.neg-edges represent interspecific competition, a higher number of Num.neg-edges implies more frequent competitive interactions between the pathogen and other bacteria. However, such redundant interactions may ultimately lead to ineffective pathogen control.

Pearson correlation analysis between soil chemical properties and co-occurrence network topological features (Figure 10) revealed significant correlations. Available potassium (AK) and available phosphorus (AP) showed significant positive correlations with most topological indices, whereas dissolved organic carbon (DOC) and pH showed significant negative correlations, These relationships indicate that microalgae application reshaped the bacterial co-occurrence network into a disease-suppressive structure—characterized by lower complexity, higher clustering, and greater modularity—through increasing DOC and pH while decreasing AK and AP, This structural shift effectively suppressed pathogen proliferation, ultimately reducing the incidence of flue-cured tobacco bacterial wilt.

**Figure 10:**
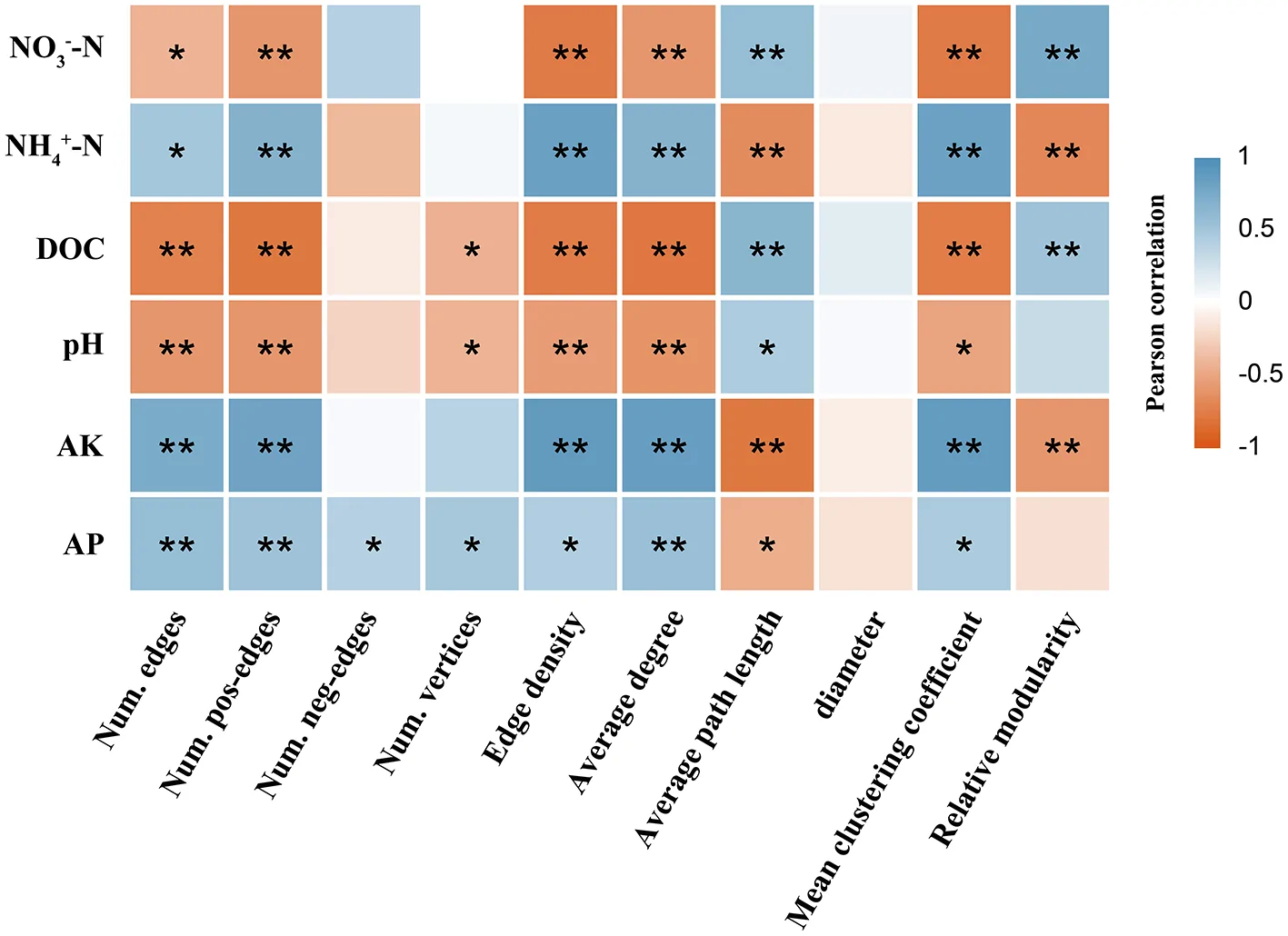
The relationship between soil chemical properties and co-occurrence networks topological features. Significance levels are as follows: *P ≤ 0.05; **P ≤ 0.01; and ***P ≤ 0.001.

## 4 Discussion

### 4.1 Microalgae application enhances tobacco yield and reduces soil pathogen abundance

In this study, microalgae application significantly reduced the incidence and disease index of flue-cured tobacco bacterial wilt while concurrently increasing tobacco yield (Figure 1). This phenotypic response was significantly correlated with the marked reduction in soil pathogen abundance (Figure 2), which is consistent with earlier reports (Renuka et al., 2016; Huo et al., 2020). Microalgae may suppress pathogen proliferation through the secretion of antimicrobial metabolites or by indirectly modifying the soil microenvironment (She et al., 2017). It has been suggested that beneficial microorganisms can inhibit pathogens via antagonism and niche competition (Compant et al., 2010), a finding that corroborates the observations of this study. The observed yield increase is a direct phenotypic consequence of reduced disease pressure. Previous studies have demonstrated that microbial inoculants can significantly enhance crop yields by controlling soil-borne diseases (Gao et al., 2019). The present study further indicates that microalgae treatments increased tobacco yield to varying degrees—by 12.2%, 17.2%, and 14.8% relative to CK—while concurrently suppressing pathogen abundance and reducing disease incidence.

### 4.2 Microalgae reduce disease incidence by altering soil chemical properties and bacterial community characteristics

Microalgal photosynthesis supplies oxygen to the soil and facilitates the decomposition of organic matter, establishing a beneficial ecological cycle that enhances microbial diversity and function in both soil and plants (Song et al., 2024). In this study, microalgae significantly increased soil dissolved organic carbon and pH, while reducing available potassium and available phosphorus. Specifically, compared with CK, the T3 treatment increased dissolved organic carbon by 15.27%, elevated pH by 1.66%, decreased available potassium by 8.69%, and reduced available phosphorus by 20.20%, which is in agreement with earlier reports Yilmaz and Sönmez, 2017. It has been suggested that soil pH and dissolved organic carbon are key drivers of microbial community structure, and that conditions with higher pH and dissolved organic carbon favor the colonization and proliferation of beneficial microorganisms (Fierer, 2017). In the present study, both pH and dissolved organic carbon were significantly correlated with microbial community structure (*p* ≤ 0.05). Previous research has indicated that a decrease in nitrate nitrogen may impair the nitrogen acquisition capacity of pathogens, and that microalgae-mediated nitrogen transformation could suppress pathogens through nitrogen competition (Yang et al., 2023). In line with this, compared with CK, T2 and T3 significantly reduced soil nitrate nitrogen at both the rapid growth stage (by 32.3% and 6.0%, respectively) and the maturity stage (by 25.6% and 19.2%, respectively). Taken together with the correlation analysis, this suggests that microalgae may have suppressed pathogen reproduction. With respect to community composition, microalgae treatments significantly altered the relative abundance of bacterial taxa at both the phylum and genus levels (Figure 6), enriching biocontrol genera such as *Sphingomonas* and *Bacillus*, while suppressing putatively pathogenic taxa including *Methylomirabilota* and *MND1*. These findings are consistent with previous observations that enrichment of specific functional groups can enhance soil disease resistance (Mendes et al., 2011). Dissolved organic carbon, comprising readily available carbon sources, can promote soil microbial metabolism (Peng et al., 2026), thereby enabling competition with plant pathogens and reducing disease incidence (Ramakrishnan et al., 2023). In the present study, the significant increase in dissolved organic carbon under microalgae treatments was accompanied by a marked reduction in pathogen abundance at the maturity stage (by 74.1%, 90.7%, and 90.7% in T1, T2, and T3, respectively), which is in accordance with previous reports.

### 4.3 Interactions between soil chemical properties and microbial community structure reduce disease incidence

Correlation analysis between soil chemical properties and dominant bacterial taxa revealed the disease-suppressive mechanism of microalgae through the integrated effects of “soil chemistry and microbial community characteristics.” It has been shown that soil pH is a key factor regulating bacterial abundance (Qi et al., 2019). In this study, compared with CK, soil pH at the maturity stage in microalgae treatments increased by 0.65%, 0.60%, and 1.66%, respectively. Along with the rises in dissolved organic carbon and pH, the abundance of biocontrol genera such as *Sphingomonas* and *Bacillus* increased significantly, while disease-promoting genera including *MND1* and *Gaiella* decreased. It has been suggested that soil chemical amendments can achieve disease control through the selective enrichment of beneficial bacteria and suppression of pathogens (Song et al., 2024; Zhang et al., 2017). In the present study, microalgae treatments increased soil pH and dissolved organic carbon while decreasing available potassium and available phosphorus at both growth stages. Such modified soil conditions were more favorable for constructing a microbial community that suppresses disease-promoting taxa and enriches disease-suppressive taxa (Solomon et al., 2023), which contributes to the development of long-term soil disease resistance, consistent with previous reports (Chen et al., 2018). Moreover, the regulation of soil chemical properties by microalgae in this study was bidirectional: it optimized chemical indicators related to carbon and nitrogen cycling (e.g., DOC, NH_4_^+^-N), while suppressing factors potentially favorable to pathogens (e.g., NO_3_^−^-N, AK, AP), in agreement with earlier observations (Kidinda et al., 2023).

### 4.4 Interrelationships among bacterial co-occurrence network topology, soil chemical properties, and disease suppression

Analysis of the topological features of bacterial co-occurrence networks (Figure 9) provided deeper insight into the disease-suppressive mechanism of microalgae from the perspective of community interactions. It has been reported that network structures with high modularity and high clustering can more effectively resist pathogen invasion (Chen et al., 2018, 2020), and that soil organic carbon content is a key chemical driver of enhanced network modularity. In this study, microalgae treatments increased dissolved organic carbon (by 6.72%, 3.19%, and 15.27% in T1, T2, and T3, respectively, relative to CK), simplified the co-occurrence network (as evidenced by reduced edge numbers and density), and concurrently enhanced network modularity and the average clustering coefficient. Soil chemical properties are recognized as important drivers of network remodeling (Liu et al., 2023). In the present study, dissolved organic carbon and pH were significantly positively correlated with network modularity and clustering (*p* ≤ 0.05), whereas available phosphorus and available potassium were significantly negatively correlated (*p* ≤ 0.05). These results suggest that microalgae, through the regulation of soil chemical properties, shifted the bacterial interaction pattern from redundant complexity to a more streamlined and efficient state. This topological optimization may impair pathogen reproduction and contribute to long-term disease control, which aligns with previous observations (Xiang et al., 2023).

In summary, this study elucidated the intrinsic relationships underlying microalgae-mediated suppression of flue-cured tobacco bacterial wilt through a multidimensional framework encompassing “phenotype – pathogen – soil chemical properties – microbial community characteristics – co-occurrence network.” Based on our findings, it is inferred that microalgae may reshape bacterial diversity, community composition, and interaction networks by modifying soil chemical properties, thereby suppressing pathogen proliferation, reducing disease incidence, and ultimately increasing tobacco yield.

## 5 Conclusions

Through a field experiment, this study elucidated the mechanism by which microalgae reduces the incidence of tobacco bacterial wilt through modifying soil properties and microbial community structure. The principal conclusions are as follows: (1) Compared with CK, yield in T1, T2, and T3 increased by 12.2%, 17.2%, and 14.8%, respectively, and disease incidence at maturity decreased by 43.7%, 18.9%, and 71.9%, respectively; (2) Pathogen abundance at maturity in T1, T2, and T3 decreased by 74.1%, 90.7%, and 90.7%, respectively, relative to CK, and soil pathogen abundance was significantly positively correlated with disease indices; (3) Microalgae treatments suppressed soil pathogen abundance and disease incidence by increasing soil pH and dissolved organic carbon while decreasing available potassium and available phosphorus; (4) Bacterial abundance at maturity in T1, T2, and T3 increased by 12%, 23%, and 33%, respectively, compared with CK, and biocontrol functional genera were enriched. Bacterial taxa at both phylum and genus levels showed significant associations with soil chemical properties and disease indices; (5) Microalgae reshaped the bacterial co-occurrence network and enhanced its modularity. In summary, microalgae suppressed pathogens and reduced disease incidence by modulating soil chemical properties, increasing bacterial diversity, enriching biocontrol genera (e.g., *Sphingomonas, Bacillus*), and optimizing the co-occurrence network. Under the experimental conditions, microalgae application at 28 mL·L^−1^ proved most effective.

## Data Availability

The raw data supporting the conclusions of this article will be made available by the authors, without undue reservation.
